# A Cross-Sectional Study on Knowledge, Awareness, Self-Care Practices About Glaucoma Among Doctors in South India

**DOI:** 10.7759/cureus.62043

**Published:** 2024-06-10

**Authors:** Adithya Tellakula, Meera Devasena M, Rejo Varkey Cyriac

**Affiliations:** 1 Ophthalmology, Sri Ramachandra Institute of Higher Education and Research, Chennai, IND

**Keywords:** healthcare professionals, self-care practice, blindness, knowledge, glaucoma

## Abstract

Introduction

Glaucoma is the major cause of irreversible blindness worldwide. Glaucoma affects the optic nerve head in the posterior segment of the eye and the defects lead to permanent blindness if left untreated. Poor knowledge about this disease is strongly correlated with delayed diagnosis. This study aimed to evaluate doctors’ knowledge and self-care practices about glaucoma at a tertiary care hospital in Chennai, presuming that healthcare professionals constitute an effective conduit between the population at risk and ophthalmologists.

Methods

We carried out this cross-sectional survey among 252 doctors practicing allopathic medicine in a tertiary care hospital in Chennai from July 2022 to December 2022. We have collected 252 samples by simple random sampling method. We have excluded doctors who have a degree in ophthalmology or practicing it. The data collection was interview-based using a pre-designed, structured questionnaire that contained questions on sociodemographic characteristics age, gender, and specialty in medicine. It also included questions assessing knowledge and self-care practices about glaucoma among doctors of various specialties other than ophthalmology. We entered the collected data into Microsoft Excel (Microsoft Corporation, Redmond, WA), cleaned it, and analyzed it using SPSS version 16.

Results

The mean age of the doctors was 33.24 ± 10.90 years in this study. About 132 (52.4%) of the study participants were females. Only 91 (36.1%) of respondents knew that glaucoma may permanently impair vision, but nearly 240 (95.2%) believed that it could be treated. Two hundred seventeen (86.1%) participants were aware that glaucoma runs in families. About 218(86.5%) doctors were aware that glaucoma destroys the optic nerve in the eye, and 171 (67.9%) knew that peripheral vision loss happens before central vision loss. Only 146 (57.9%) of physicians had their eye pressure examined. About 232(92.1%) doctors had their eyes checked routinely. Only 42 (16.7%) of physicians took part in glaucoma awareness campaigns. Nearly 199 (79%) of those surveyed thought it was required to check a patient’s family members for glaucoma. We note that among doctors, the knowledge and practice score of correct replies was strongly connected with work experience (P value = 0.035).

Conclusion

The need for extensive eye health education and information distribution for healthcare workers should be stressed. Teaching the hospital staff about the symptoms and prognosis of this “silent thief of sight” might be a crucial first step in providing preventive ophthalmic treatment.

## Introduction

Glaucoma is an eye disease that gradually disrupts visual function and harms the optic nerve head and results in irreversible blindness. Glaucoma is defined as chronic progressive optic neuropathy with structural irreversible optic nerve head changes and functional visual field defects with increased or normal intra-ocular pressure. As retinal ganglion cells cannot regenerate and due to gradual ganglion cell loss, the conducting function of the optic nerve is compromised that leads to permanent vision loss. These ailments come in both acute and chronic forms, affecting both adults and children [1]. Glaucoma-related visual impairment negatively affects a person’s social and economic well-being [2]. The major cause of permanent blindness worldwide is glaucoma. Currently, there are around 60 million glaucoma sufferers globally, and by 2040, it is projected to rise to 110 million [3].

Glaucoma is the primary cause of irreversible blindness in India, affecting at least 12 million people and leaving approximately 1.2 million of them legally blind. More than 90% of glaucoma cases in the general population go undetected [4]. About 5.80% of India’s total burden of avoidable blindness is attributable to glaucoma. A significant part of India’s national goal to control blindness is preventing glaucoma-related blindness [5]. According to the adverse childhood experiences (ACES) research, the prevalence of primary open-angle glaucoma (POAG) among people aged 40 and over in South India was estimated to be 1.7% in the rural population and 3.5% in the urban population [6,7]. Early diagnosis and treatment are the most effective methods for preventing glaucoma-related blindness. Diagnoses are frequently made after the illness has already severely damaged the optic nerve function in the eye [8]. Delay in diagnosis was strongly correlated with inadequate glaucoma knowledge [9-11]. Numerous population-based studies have shown that glaucoma awareness and knowledge are astonishingly low among both rural and urban populations, mainly in developing nations, which has an adverse effect on health-seeking measures [12]. When compared to other disorders like cataracts (69.8%), night blindness (60%), and diabetic retinopathy (27%), glaucoma awareness in the general community was very low (2.3%), which has a detrimental effect on the behavior of people who seek out healthcare [13]. Because glaucoma is linked to comorbidities, healthcare workers have a crucial role in connecting patients with risk factors to ophthalmologists for screenings and in raising awareness about the disease [14]. Given that the reach of the healthcare system throughout developing nations is still far from ideal, it is crucial that all healthcare professionals get adequate education about glaucoma blindness to reach a substantial portion of the population at risk of gaining access to a comprehensive eye care facility.

Aim

This study was designed to evaluate the knowledge and self-practice of healthcare workers on glaucoma at a tertiary care hospital in Chennai, South India presuming that they represent a crucial link role in connecting general patients at risk to eye specialists for screenings and in raising awareness about glaucoma.

## Materials and methods

Study methodology

This study is an analytical cross-sectional study, carried out with a questionnaire-based survey among doctors practicing allopathic medicine in various specialties in tertiary care hospitals in Chennai from July 2022 to December 2022. We have included all physicians and surgeons practicing allopathic medicine currently working in a tertiary care hospital in Chennai. We have excluded doctors who have a degree in ophthalmology and practicing it.

Sample size and sampling technique

Considering previous data showing that the prevalence of knowledge in Nigeria [15] was 36.8%, with a 95% confidence level and 6% allowable error, we estimated the minimum sample size to be 249 doctors. We estimated the sample size using the formula n= Z21- α/2 PQ/d2 (Z=1.96, P=36.8, Q=63.2, d=6). We have collected information from 252 doctors by simple random sampling method. We have developed a sampling frame by obtaining the name list of doctors from the administration. Before conducting the study, we obtained an institutional ethical clearance certificate for the study (approval number: CSP-MED/21/JUL/70/103). We explained the objectives of the study to participants before they gave their consent to participate.

Data collection procedure

The questionnaire was developed based on the information obtained from similar studies related to the topic and was pretested among 20 doctors working in other hospitals. Based on the observations of pretesting, the questionnaire was modified and validated by experts in ophthalmology and community medicine. The validated questionnaire was then used for the data collection in the final study with questions on age, gender, and medical specialization which was interview-based. It had inquiries evaluating clinicians’ self-care practice and comprehension of glaucoma. This study tool had questions and multiple-choice answers like “yes,” “no,” and “don’t know,” in English. The scoring system for awareness and knowledge questions was a score of 2 for a correct response, 1 for an incorrect response, and a score of 0 for don’t know response. In order to know the importance, weightage was given for these variables as 2, 1, and 0, for awareness and knowledge-based responses; however, during analysis, it was recorded as a score 1 for a correct response and score 0 for the other responses. The scoring of self-care practice responses are score 1 for “Yes” and 0 for “No”.

Data analysis

The scored variables were recorded and analyzed by the statistician. We entered the collected data into Microsoft Excel (Microsoft Corporation, Redmond, WA) and analyzed it using SPSS version 16 (IBM Corp., Armonk, NY). To summarize the sociodemographic characteristics of the study population, we employed variables, and we used the independent ‘t’ test to look for associations with the outcome variables, such as knowledge and self-care practice. To forecast knowledge and practice from the job experience element, we employed Pearson’s correlation.

## Results

The mean age of the doctors was 33.24 ± 10.90 years in this study. About 132 (52.4%) of the samples were females. Among the participating doctors 32 (12.7%) of the respondents belong to the general medicine department followed by 25 (9.9%) from the anesthesia department. We have described the basic characteristics of the study participants in Table 1.

**Table 1:** Basic characteristics of the study participants *Mean ± standard deviation

Variable		Frequency (%)
Age		33.24 ± 10.90*
Gender	Male	120 (47.6)
	Female	132 (52.4)
Speciality	Anesthesia	25 (9.9)
	Community medicine	21 (8.3)
	General medicine	32 (12.7)
	General surgery	18 (7.1)
	Resident doctors	22 (8.7)
	Obstetrics and gynecology	7 (2.8)
	Orthopedics	12 (4.8)
	Pediatrics	18 (7.1)
	Others-medical officers, etc.	97 (38.5)

We have described the responses of doctors to questions assessing the knowledge of glaucoma in Table 2. All the study participants were aware of the disease glaucoma in the eye. A total of 240 (95.2%) participants agreed that glaucoma is treatable but only 91 (36.1%) were aware that blindness from glaucoma is irreversible. About 217(86.1%) participants had an understanding that glaucoma runs in families and 227 (90.1%) had an awareness that glaucoma can occur at any age. A total of 166 (65.9%) doctors believed that glaucoma does not always occur in both eyes. About 218 (86.5%) among the 252 participants had an understanding that glaucoma damages the optic nerve in the eye and 171 (67.9%) had knowledge that vision loss first occurs in the periphery rather than central. A total of 177(70.2%) doctors understood that vision disturbance in the early stage is unnoticeable. Only 30 (11.9%) doctors had a familiarity that glaucoma is predominantly an asymptomatic disease leading to permanent blindness.

**Table 2:** Responses of glaucoma-aware participants to questions to assess knowledge of glaucoma

Awareness Questions	Yes	No	Don't know
	N (%)	N (%)	N (%)
Can anyone have glaucoma?	230 (91.3)	12 (4.8)	10 (4.0)
Is glaucoma treatable?	240 (95.2)	12 (4.8)	0 (0.0)
Blindness from glaucoma is reversible.	130 (51.6)	91 (36.1)	31 (12.3)
Glaucoma can run in families.	217 (86.1)	12 (4.8)	23 (9.1)
Glaucoma can occur at any age.	227 (90.1)	19 (7.5)	6 (2.4)
Glaucoma always affects both eyes.	59 (23.4)	166 (65.9)	27 (10.7)
Glaucoma is related to eye pressure.	249 (98.8)	3 (1.2)	0 (0.0)
Glaucoma damages optic nerve in the eye.	218 (86.5)	17 (6.7)	17 (6.7)
Vision disturbance in the early stage is noticeable.	60 (23.8)	177 (70.2)	15 (6.0)
Vision loss first occurs in the peripheral vision.	171 (67.9)	37 (14.7)	44 (17.5)
Glaucoma can be related to long-term steroid use.	207 (82.1)	10 (4.0)	35 (13.9)
Glaucoma is always symptomatic.	195 (77.4)	30 (11.9)	27 (10.7)
Available treatments are medical, surgical, and laser.	159 (63.1)	85 (33.7)	8 (3.2)

We have described the responses of doctors to questions assessing the self-care practices of glaucoma in Table 3. About 232 (92.1%) participant doctors had done their eye checkup but only 146 (57.9%) had checked their eye pressure. Only 42 (16.7%) doctors had participated in glaucoma awareness programs. About 199 (79%) of the respondents believed that screening of family members of a glaucoma patient is mandatory.

**Table 3:** Responses of glaucoma-aware participants to questions to assess self-practices of glaucoma

Self-Practices Questions	Yes	No
	N (%)	N (%)
Have you undergone an eye checkup?	232 (92.1)	20 (7.9)
Have you checked your eye pressure?	146 (57.9)	106 (42.1)
Is screening of family members of glaucoma patient mandatory?	199 (79.0)	53 (21.0)
Glaucoma treatment and follow-up are lifelong.	223 (88.5)	29 (11.5)
Have you participated in glaucoma awareness programs?	42 (16.7)	210 (83.3)

We have described the assessment score of awareness and self-practice among the study samples in Table 4. The study participants had good knowledge regarding glaucoma, but self-practice needs to be improved as it is moderate.

**Table 4:** Assessment score of awareness and self-practice among the study samples

Assessment Score	Minimum	Maximum	Mean ± Standard deviation
Total score of the questionnaire	14	40	30.47 ± 5.19
Knowledge score	2	12	9.45 ± 1.75
Practice score	0	5	3.34 ± 1.09
Knowledge and practice score of correct responses	6	17	12.79 ± 2.08

We used an independent “t” test to find out the association between gender and the assessment score of study participants. From the test, we inferred that knowledge and self-practice score as well as total score of questionnaires and correct responses was not influenced by gender where the P value of parameters was greater than 0.05 which is explained in Table 5.

**Table 5:** Association between gender and assessment score of study participants by using independent t test

Assessment Score	Gender	Frequency	Mean ± Standard Deviation	P value
Total score of the questionnaire	Male	120	30.37 ± 5.44	0.767
	Female	132	30.56 ± 4.98	
Knowledge score	Male	120	9.44 ± 1.83	0.926
	Female	132	9.46 ± 1.67	
Practice score	Male	120	3.31 ± 1.21	0.651
	Female	132	3.37 ± 0.96	
Knowledge and practice score of correct responses	Male	120	12.75 ± 2.20	0.751
	Female	132	12.83 ± 1.97	

We used Pearson’s correlation test to find out the correlation between work experience and the assessment score of study participants (Table 6). From the test we inferred that work experience was strongly correlated with self-care practice among doctors (P value = 0.0005) and work experience was positively correlated with knowledge and practice score of correct responses among doctors (P value = 0.035).

**Table 6:** Correlation between work experience and assessment score of study participants by using Pearson correlation test *Correlation is significant at the 0.05 level (2-tailed) **Correlation is significant at the 0.01 level (2-tailed)

Variable	Value	Total Score of Questionnaire	Knowledge Score	Practice Score	Knowledge and Practice Score of Correct Responses
Work experience (after UG) in years	r-value	0.081123	-0.00642	0.264**	0.133*
	P value	0.199316	0.919233	0.0005	0.035212
	N	252	252	252	252

## Discussion

In this study, only 36.1% of participants were aware that blindness from glaucoma is permanent. Research by Nageeb and Kulkarni involving 114 medical specialists in Mangalore, India, determined that their understanding of the relationship between glaucoma and elevated IOP (82.40%) was superior to optic nerve injury (32.40%) [14]. Although working in a hospital with simple and free access to healthcare, only 57.9% of doctors in our study had their eyes tested for eye pressure, compared to just 15.7% of doctors in the study by Nageeb and Kulkarni [14].

In this study, about 86.1% of doctors understood that glaucoma runs in families and 82.1% of them had sufficient information that glaucoma can be associated with long-term steroid usage. In the study done by Nageeb and Kulkarni, 83.3% of doctors knew that corticosteroids were a risk factor for glaucoma, and 94.4% of them knew that glaucoma runs in families [14]. Because clinicians are aware of the negative consequences of long-term steroid use, they have appropriate knowledge regarding steroid use in both trials.

In this study, nearly 98.8% of participants knew that high intraocular pressure was a contributing factor to glaucoma and that glaucoma affects the optic nerve in the eye. Similar results were seen in the African study by Komolafe et al. in which 88.3% of experts were aware that glaucoma was caused by excessive ocular pressure [16]. In our study, almost 99% of participants are aware that glaucoma is related to eye pressure whereas it was just 39% in a study by Adegbehingbe and Bisiriyu in Nigeria [17].

Work experience and the correct response rate among the participating doctors were positively associated with their higher scores in this study. These individuals could have easier access to pertinent information due to their greater level of education. The substantial relationship between educational achievement and work experiences that was also seen in this study may have contributed to the correlation between occupation and knowledge of glaucoma.

Although having a sufficient comprehension of the disease is important, having a good awareness of glaucoma does not imply that the subject is an expert on the condition. As a result, efforts to lessen the burden of sickness will be ineffective if they are made without raising the level of awareness among medical professionals.

Limitation

Doctors from tertiary care facilities participated in this hospital-based single-center study, therefore it might not accurately reflect the situation in the communities. A teaching hospital’s doctors may have greater knowledge and awareness than the general population.

## Conclusions

Based on the results, we draw the conclusion that doctors in departments other than ophthalmology are frequently the first people patients contact when seeking medical advice. These medical professionals must be knowledgeable, or the risk of providing patients with incorrect information and unhelpful counseling is very high, even in tertiary care facilities. It must be remembered that it is wrong to believe that non-ophthalmologists have enough understanding of and a suitable attitude toward glaucoma. The likelihood that medical professionals will check themselves and their family members for glaucoma will rise if there is a general increase in medical professional knowledge of glaucoma. This trend will progressively convert into a culture of frequently recommending patients for glaucoma screening, ultimately leading to an increase in the early identification of glaucoma and thus preventing blindness.
